# Communication in Paediatric Oncology: A Qualitative Study Exploring the Perspectives of Nurses and Parents of Children With Cancer

**DOI:** 10.1002/nop2.70371

**Published:** 2025-11-19

**Authors:** Reza Saidi, Haydeh Heidari

**Affiliations:** ^1^ School of Nursing and Midwifery Shiraz University of Medical Sciences Shiraz Iran; ^2^ Faculty of Nursing and Midwifery, Modeling in Health Research Center Shahrekord University of Medical Sciences Shahrekord Iran

**Keywords:** cancer, communication, nurses, paediatric oncology, parents

## Abstract

**Aim:**

The present study explored the perspective of nurses and parents of children with cancer regarding communication.

**Design:**

This was a qualitative study that applied a descriptive‐exploratory approach.

**Methods:**

Participants included six nurses, eight mothers and four fathers of children with cancer, who were selected based on a purposive sampling method. Data were collected using in‐depth interviews with participants. All interviews were recorded and transcribed verbatim. Data were analysed using the inductive content analysis method.

**Results:**

Data analysis revealed three categories and eight subcategories, including supportive communication (subcategories: informational support, psychological support and special communication skills); managing spiritual crisis (subcategories: identification of spiritual/religious beliefs and needs of children with cancer and their parents, and managing the spiritual crisis of children with cancer and their parents); and empowering parents and healthcare teams (subcategories: the necessity of teamwork, holding educational workshops and training paediatric oncology nurse specialists). All healthcare providers (including nurses, oncologists, paediatricians, etc.) can consider the results of this study to improve their communications with other healthcare team members, children with cancer, and their families to provide holistic care and increase the quality of care they provide.

**Patient or Public Contribution:**

No patient or public contribution.

## 1 Introduction

Cancer is a rare disease in people younger than 20 years old (Steliarova‐Foucher et al. 2017); however, childhood cancers are among the leading causes of morbidity in patients younger than 14 years old in several countries worldwide (Vos et al. 2016). The incidence of childhood cancers varies among different nations and races and depends on various factors such as genetic predisposition and exposure to infectious or environmental carcinogens (Khazaei et al. 2017). Approximately each year, 75–150 new cases of paediatric cancers are identified per one million people worldwide (Siegel et al. 2018). In Iran, it has been reported that the age‐standardised incidence rate of all‐cause cancers in children younger than 15 years old is 11.9 new cases per hundred thousand people (Shabani et al. 2020).

Cancer diagnosis causes significant psychological stress to patients and their family members. If these patients are children and adolescents, this stress will be more severe (Mohammadzadeh et al. 2022). On the other hand, the process of treating paediatric cancer may continue for several years. This process requires various medical care, both at home and in the hospital, and parents of children play an essential role in caring for the child. These parents have a crucial role in chemotherapy, maintaining the oral and dental health of the child undergoing chemotherapy, managing the oral side effects of prescribed drugs, maintaining the hygiene of the venous access ports (such as the chemotherapy port), monitoring the side effects of treatments and the general health status of their child (Clarke and Fletcher 2003). Thus, when a child is diagnosed with cancer, their parents experience significant distress and face lots of information and new responsibilities. This distress can disrupt parents' ability to process and use information, make informed decisions about the child's treatment process, and continue the required care at home (Sisk, Keenan, Blazin, et al. 2021). This distress can also reduce parents' ability to manage time and deal with existing conditions, which eventually causes problems in their mental health. Parental distress can also harm the relationship between the child and the parents, which ultimately causes psychological complications in the child with cancer (Cowfer et al. 2021).

The intense distress experienced by parents of children with cancer can disrupt the process of establishing communication between healthcare professionals, children and parents (Epstein and Street Jr 2007; Fallowfield and Jenkins 2004; Moore et al. 2018). It should be noted that, from the time a child is diagnosed with cancer until the time of complete cure or the child's death, effective communication between the healthcare professionals, the child and his/her parents is crucial and has several vital roles (Sisk, Mack, et al. 2018). Such communication can reduce the distress experienced by parents of children with cancer and help them take care of their children. This communication can also be associated with parents' peace of mind (Mack et al. 2009), increased parents' hopefulness (Mack et al. 2007; Nyborn et al. 2016), increased trust in the healthcare professionals (El Malla et al. 2013; von Essen et al. 2001), the feeling of being approved (Arabiat et al. 2011), and a sense of comfort in parents (Young et al. 2013). Effective communication can also support parents in decision‐making (Mack et al. 2011).

Therefore, establishing proper communication with patients and family members is one of the main goals of the paediatric nursing profession (Hopia and Heino‐Tolonen 2019). Nurses caring for children with cancer should provide psychological, social and holistic care to patients and their family members. Thus, establishing proper communication with children with cancer and their family members is particularly important (Hentea et al. 2018; Newman et al. 2020). However, establishing such communication is complex and challenging (Sisk, Mack, et al. 2018; Sisk, Schulz, et al. 2019). Results of various studies show that in many cases, healthcare professionals and nurses have not succeeded in establishing effective communication with children with cancer and their families (Mack et al. 2011; Rosenberg et al. 2014; Sisk, Kang, et al. 2019; Sisk et al. 2017; Sisk, Kang, and Mack 2018).

Establishing effective communication between healthcare professionals, children with cancer, and their family members may fail for various reasons, including the necessity of sharing the bad news, problems related to determining the most appropriate time to provide information to the parents, parents' unpreparedness for communication with healthcare professionals, uncertainty about the future, and the use of specialised medical terms by the healthcare professionals (Brouwer et al. 2021; Sisk, Friedrich, Kaye, et al. 2021). Furthermore, parents of children with cancer have expressed various obstacles to effective communication with their child and healthcare professionals, which include the desire to protect their child against fear and other unpleasant feelings, the idea of imposing a lot of psychological burden on the child, being concerned that their child loses trust in parents, fear of the child's reaction, feeling too much tension, and lack of self‐confidence for establishing communication (Cowfer et al. 2021). Failure to develop effective therapeutic communication between healthcare professionals, children with cancer, and their family members may cause parents to feel their need for information is not adequately met, regret their decisions, lose their trust in healthcare professionals, and have less adherence to prescribed treatments (Sisk et al. 2022).

It is also important to note that communication between children with cancer, their parents, and healthcare professionals is a multifaceted process, and each of the parties may have a different interpretation of the concept of communication. These differences in opinions about the concept and importance of communication may cause misunderstandings, feelings of disappointment, and uninformed decisions (Sisk, Schulz, Kaye, et al. 2021). Therefore, it seems necessary to explore the experiences and perspectives of nurses and parents of children with cancer regarding the concept of communication and its challenges. We found no study investigating this issue in Iranian society. Iranian society comprises people with various ethnicities and cultural backgrounds (e.g., Arab, Baloch, Gilaki, Kurdish, Lor, Mazani, Persian, Tork, Turkmen…) and even multiple religions. In this regard, Iranian nurses have to communicate with clients with different ethnic and cultural backgrounds (Zeidani et al. 2023). Since communication in the paediatric oncology unit is critical, it is necessary to identify the experiences of parents and nurses to identify possible challenges and issues in this regard. Thus, the current study aimed to investigate the perspectives of nurses and parents of children with cancer about communication, along with its challenges and issues. This aim is covered in the following research question: How do nurses and parents of children with cancer experience communicating in the oncology unit?

## 2 Methods

### 2.1 Study Design

This is a qualitative study which applied a descriptive‐exploratory approach. This approach is used to evaluate the phenomena related to health care and nursing. Descriptive‐exploratory qualitative studies are used when there is not enough information and knowledge about a subject or phenomenon, and this particular subject or phenomenon can only be identified by evaluating the opinions and experiences of the people involved and related to that subject (Kim et al. 2017).

### 2.2 Study Settings and Population

Participants in this study included parents of children with cancer hospitalised in the oncology unit at Ayatollah Kashani Hospital and their nurses in 2023. The age of children was 4–12 years old diagnosed with one of the cancers listed in the International Classification of Childhood Cancer (ICCC).

### 2.3 Inclusion and Exclusion Criteria

Inclusion criteria for nurses were: willingness to participate in the study, having at least a bachelor's degree in nursing, working in an oncology unit for at least 6 months, and having experience in caring for children with cancer. The inclusion criteria for parents of children with cancer were the willingness to participate in the study. The exclusion criterion was the unwillingness to cooperate at any study stage. It should be noted that no participants were excluded from the study.

### 2.4 Sampling Procedure

This study used a purposive sampling method to select participants with rich information regarding the importance and challenges of communication in paediatric oncology settings. Sampling continued until reaching data saturation. Data saturation happens when interviews with new participants yield no new information or when participants provide repetitive data already noted by the previous participants (Lincoln 1985). In the present study, data saturation was reached after interviewing 18 participants.

### 2.5 Data Collection

In the present study, data were collected using semi‐structured interviews with nurses and parents of children with cancer. At the first step of data collection, the researcher (H.H.) conducted some preliminary semi‐structured interviews with participants (nurses and parents) to get familiar with the possible and unforeseen issues and form the questions' composition. These interviews had no fixed and predetermined questions, and the study questions were formed based on these interview processes. After these interviews, the study questions were formed, which are presented in Table 1.

**TABLE 1:** The interview guide.

participant	Example semi‐structured interview guide	Probing question
Nurses	– Could you please tell us about your experience working with children with cancer and their parents? – What problems did you face during your communication? – What strategies do you suggest to improve communication between children with cancer and their parents?	– Can you explain more? – Please give me an example – Please give me more information
Parents	– Tell us about experiences regarding communication with nurses – Tell us about experiences regarding communication with your child – What strategies do you suggest to improve communication with nurses and your child?	– Can you explain more? – Please give me an example – Please give me more information

In the next step, main interviews with participants were performed (by H.H.). First, the interview was started with open‐ended questions (Table 1). Interviews continued with probing questions such as please explain more. After conducting several interviews with participants and gathering new ideas, the researcher (H.H.) reviewed the interview questions again and found no need to modify or change them. In general, the duration of each interview depended on the interviewee, the topic of the interview, and the method used, and was between 30 and 60 min. Interviews were conducted in a calm environment, and the participants' comfort was considered. During participant interviews, the researcher (H.H.) used observation and field notes to collect information. Each participant was interviewed once, and there was no need to repeat the interview with any participants. All interviews were recorded entirely using an MP3 recorder device. All recorded interviews were transcribed verbatim, and their contents were analysed and coded.

### 2.6 Data Analysis

Data were analysed using inductive content analysis. The inductive content analysis process includes open coding, classification and abstraction (Elo and Kyngäs 2008). Analysis units were parts of transcribed texts of interviews that were related to the study objectives (i.e., communication issues and challenges). After selecting analysis units, data analysis began with repeated text reading to immerse in the data and find an overview.

For open coding, the researcher extracted data (preliminary ideas) from transcribed texts of interviews. This process involved repeatedly reading each interview, during which all relevant codes related to the study's objectives were written in the margin of the texts as notes. In the next step, the researcher listened to recorded interviews again to ensure no critical point was missed. The data (ideas) obtained in the open coding process were organised into meaning units, and each meaning unit was assigned a specific code for identification. An example of open coding is presented in Table 2.

**TABLE 2:** Example of meaning unit, open coding, subcategories and category.

Meaning unit	Open coding	Subcategories	Category
I searched the Internet and got some information. The doctor talked to us about treatment and chemotherapy	Information searching	Informational support	Supportive communication
But I don't know anything about care and the possibility of recovery	Need to support		

**FIGURE 1:**
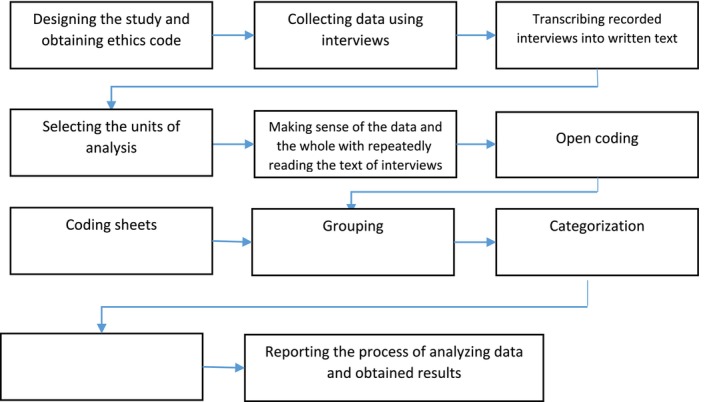
Study flowchart.

In the classification step, extracted codes were classified into categories and related subcategories based on their differences and similarities. The abstraction step was conducted after categorisation to formulate a general description of the research topic. To perform abstraction, each subcategory was assigned a defining term. Subcategories with similar ideas were then grouped into categories, and categories were further organised into main categories. The flowchart of the study process is presented in Figure 1.

### 2.7 Trustworthiness

Four criteria, including credibility, dependability, transferability and confirmability, are suggested by Speziale et al. (2011) to ensure the strength of qualitative research. These four criteria are applied to the methodology of the present study to ensure the trustworthiness of the findings and are described below:

#### 2.7.1 Credibility

In qualitative studies, credibility means evaluating extracted information in terms of accuracy (Speziale et al. 2011). In the current study, the following methods were used to increase the credibility of the data: conducting in‐depth interviews in multiple sessions, combining different methods of data collection such as interviews, field notes and observation, asking participants to review data and extracted codes and confirm or correct the codes, asking experts to evaluate extracted codes to ensure that identified categories match the statements of the participants, specifying the researcher's ideas and presuppositions to prevent their impact on data analysis (bracketing). It is important to note that the participants and experts who reviewed the obtained results did not request any changes and approved the findings.

#### 2.7.2 Dependability

Dependability means that the readers can evaluate the adequacy of analyses by following the researcher's decision‐making steps. In this study, to ensure the dependability of results, faculty members of the Nursing School of Shahrekord University of Medical Sciences who had experiences and expertise in qualitative research were asked to evaluate the data analysis process and obtained results and tell whether they are accurate or not. They approved the study process and its results without requests for modifications.

#### 2.7.3 Transferability

Transferability is similar to generalizability in quantitative research and means that the findings of the study can be transferred to similar situations or participants. Techniques such as the purposive sampling method, interviewing with various participants, and using direct quotes and examples in interviews, were applied in this study to increase the transferability of results. In addition, the obtained results were presented to some people with the characteristics of the participants (who did not participate in the research) to judge the similarity between the study results and their experiences.

#### 2.7.4 Confirmability

The research team kept all documents and data during all stages of the research to ensure the confirmability of results. Furthermore, some interviews and extracted codes and categories were provided to academic faculty members who had no role in the data analysis process to evaluate the accuracy of data coding, and they approved this process.

### 2.8 Ethical Considerations

The present study is approved by the Research Ethics Committees of Shahrekord University of Medical Sciences’ (ethics code: IR.SKUMS.REC.1402.069). Before starting interviews, an informed consent form was obtained from all participants, and the aims and protocol of the study were described to all participants. Permission for recording interviews was obtained from all participants. All participants were encouraged that their participation in the research was optional and that they could leave the research at any time.

## 3 Results

**FIGURE 2:**
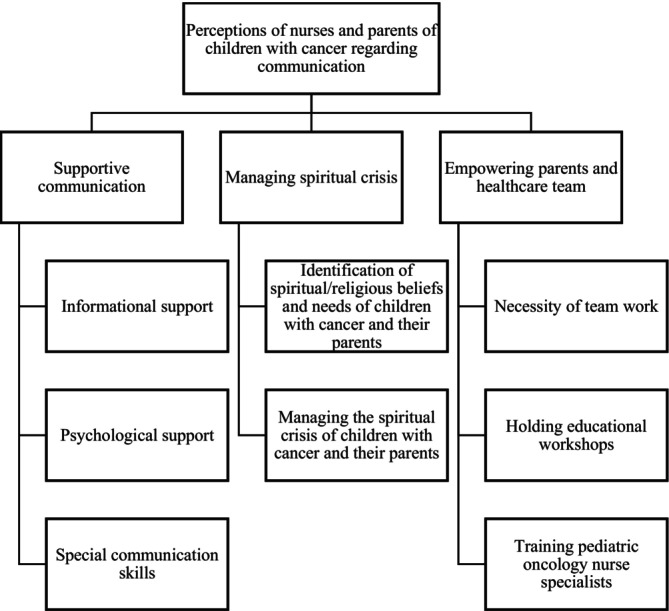
Categories and subcategories emerged in the study.

Six nurses, eight mothers and four fathers of children with cancer participated in this study. Participants' demographic characteristics are shown in Table 3. After analysing the data, three main categories emerged, including ‘supportive communication’, ‘managing spiritual crisis’ and ‘empowering parents and healthcare team’ (Figure 2). These categories and related subcategories are described below.

**TABLE 3:** Demographic characteristics of participants.

Participant no	Age	Gender	Education	Work experience (year)
M1	29	Female	Diploma	—
M2	32	Female	Bs	—
M3	40	Female	Bs	—
M4	32	Female	Diploma	—
M5	44	Female	Bs	—
M6	34	Female	Bs	—
M7	29	Female	High school	—
M8	36	Female	Bs	—
F1	35	Male	Diploma	—
F2	37	Male	Bs	—
F3	40	Male	Diploma	—
F4	41	Male	Bs	—
N1	46	Female	Bs	2
N2	48	Male	Bs	5
N3	43	Female	Bs	4
N4	27	Male	Bs	4
N5	31	Female	Bs	3
N6	38	Female	Bs	6

### 3.1 Category 1: Supportive Communication

This category included three subcategories: informational support, psychological support and special communication skills.

#### 3.1.1 Informational Support

In most cases, parents of children with cancer stated that their cancer information is insufficient, and such a feeling can cause uncertainty, anxiety and depression for them. A mother with a 4‐year‐old child diagnosed with leukaemia (6 months after diagnosis) said:

It's hard to hear the word cancer. When the doctor first told me that my child has leukemia, I had no idea. I searched the Internet and got some information. The doctor talked to us about treatments and chemotherapy, but I don't know anything about care and the possibility of recovery. I want doctors or nurses to explain the duration of treatment and care. [M3]

A father of a 10‐year‐old child with Hodgkin's disease said:

I was a clothing seller, and I had no knowledge of Hodgkin's disease, which is my child's disease. I only knew that cancer is a disease without a cure, and I really wanted the nurses to explain it to me. [F2]

According to the participants' quotations, nurses can meet the informational needs of parents of children with cancer by establishing proper communication.

#### 3.1.2 Psychological Support

In many cases, parents of children with cancer said that the distress caused by caring for a sick child increases to such an extent that it has tremendous effects on their physical, mental and social health. Most of these parents admitted in all their statements that all aspects of their family life are affected by their child's disease.

Parents who participated in this study stated that they indeed have many expectations and dreams for their child's future, but this future is suddenly questioned by suffering from a severe disease like cancer. Furthermore, when a child is diagnosed with cancer, the life situation of all family members changes drastically. The child must prepare him/herself for a long and painful treatment period. In addition, the child will be away from his family, kindergarten or school, and friends. This separation imposes a heavy psychological burden on children, parents and siblings. Thus, the children and their families need care with compassion, empathy and psychological support from the healthcare team to manage this difficult situation. In such situations, establishing effective communication between the healthcare team and parents of children with cancer is crucial.

A father with a 7‐year‐old child 3 months after diagnosis of Hodgkin's disease stated:

There are many problems related to the disease. My child has no appetite. After chemotherapy, he had nausea and fever. We did not know what to do. Even though the doctors give medications, we still have many problems. My child's hair loss is another problem, and my child was terrified to see it. We have fears and worries and do not want to be disappointed. We are wandering, and our lives are paralyzed. We feel alone … receiving support from doctors and nurses is very important. When they communicate, listen to, and empathise with us, we feel better. [f3]

A mother whose child was diagnosed with leukaemia 2 years ago expressed:

The thought of losing my child is always with me; when this thought becomes intense and the problems increase, I have no patience anymore. … Due to the chronic nature of my child's disease, we need help and support from nurses and doctors. They should hear our concerns and respond as much as possible. [m2]

A nurse said:

I have 6 years of experience in pediatric oncology. There were moments of physical and mental pain! A nurse should be full of love for service like a mountain so that he/she can withstand the pain and suffering of children and their families, like steel, and be the pacificator of their pains when communicating with them. Yes! I am speaking about nurses who care for children with cancer and are drowned in the terrible vortex of cancer. The earthly angel who is, with their loving hands and kindness and compassion, are like butterflies around the dim candle of their patients. [N6]

Based on the analysis of the data, it was found that parents of children with cancer need psychological and emotional support from healthcare providers, and this support could be provided through establishing effective, therapeutic communication.

#### 3.1.3 Special Communication Skills

*Most* participants said *that* children with cancer and their families have special information and care needs regarding various treatments, prognosis, home care, managing side effects of treatments and so on. They also experience significant pain and suffering. In this regard, nurses should have specific skills for providing high‐quality care for children with cancer and their families. One of these skills is communication skills which must encompass the ability to provide appropriate information, be kind, and provide maximum possible comfort for patients and their families. In addition, establishing effective communication with children in any setting requires specialised skills.

A nurse with 3 years of experience in paediatric oncology stated:

We need more love and special communication skills in this profession to communicate with children who are fighting a difficult disease called cancer. Children are innocent, and when we care for them, we must have sufficient knowledge and specialized skills to communicate with them properly. When this communication is established, the child cooperates to take intravenous or oral medications. [N5]

A mother with a 3‐year‐old child diagnosed with leukaemia 3 years ago said:

Due to the long period of hospitalization for children with cancer, who are in great need of love and help, the hospital has hired experienced nurses in this unit. The nurses are enthusiastic and good, and they understand us and behave well with my child… for the parents of children, the oncology unit is their second home, and the good behaviour of the doctor and nurses has a positive effect on my and my child's peace…. [m8]

Based on participants' statements, nurses in the paediatric oncology unit need special communication skills so they can meet all the needs of children with cancer and their families.

### 3.2 Category 2: Managing Spiritual Crisis

This category includes two subcategories: identification of spiritual/religious beliefs and needs of children with cancer and their parents and managing the spiritual crisis of children with cancer and their parents.

#### 3.2.1 Identification of Spiritual/Religious Beliefs and Needs of Children With Cancer and Their Parents

Parents who participated in this study expressed that their children have experienced fear, anxiety, and insomnia after being informed about the diagnosis. They bite their nails, they get afraid when they get close to the hospital, they are scared of sitting in a wheelchair and losing their hair, and they ask their mother for wigs. All these mentions clearly show their fear and anxiety during this period. Participants in this study also stated that being religious/spiritual and doing religious activities, such as praying and communicating with God, helps them cope with the difficult situation caused by the child's disease. So, the identification of spiritual/religious beliefs of children with cancer and their parents is crucial for all healthcare professionals.

A father with a 4‐year‐old child diagnosed with leukaemia said:

…Nurses respect the patients' religious beliefs. Nurses need to identify families' religious beliefs because praying calms us down. I think that no one but God can help us in this situation, but my child asks difficult questions. For example, my son used to tell me that if I die, I will go to hell or heaven; I don't know what answer I should give…. [f4]

A nurse with 4 years of experience in the oncology unit stated:

Nurses must identify the religious beliefs of parents and children. Over the years, I have noticed that parents with a stronger religious/spiritual background are less likely to have a spiritual crisis because they believe God is always with people and supports them. The relationship with God and being religious is important for coping with the disease and being hopeful. [n3]

#### 3.2.2 Managing the Spiritual Crisis of Children With Cancer and Their Parents

Parents of children with cancer are faced with philosophical questions from children, and on the one hand, as supporters, they have to connect their children to a supreme power to help them reach inner peace. In addition, spirituality and religious beliefs have an impact on children's perception of illness, suffering and issues related to it. The main concerns of parents participating in this study were philosophical questions about life, illness and death that children ask. They stated that their children ask them questions about why they got sick and also questions about death, life after death, heaven, and hell. In this regard, nurses should help children with cancer and their parents find the best answers to their spiritual/religious questions.

A mother with an 11‐year‐old child diagnosed with leukaemia said:

My child asks me why am I sick? Why am I sick among my friends and relatives' children? Why are all the bad things for me? Or how am I going to die? What happens after death? Do I see you in the other world? … [m8]

A nurse with 5 years of experience in the oncology unit expressed that:

The main concern of parents is to answer children's philosophical questions about death, why did I get sick, am I going to die? Parents have asked me to answer these questions frequently, but I didn't know. What answer should be given to such children…. [n2]

As the data above indicate, nurses can identify the spiritual needs of children with cancer and their families, as well as manage the spiritual crises these families may encounter, by establishing proper communication.

### 3.3 Category 3: Empowering Parents and Healthcare Team

This category had three subcategories: the necessity of teamwork, holding educational workshops and training paediatric oncology nurse specialists.

#### 3.3.1 The Necessity of Teamwork

Children with cancer and their families have various needs in different aspects of life, including physical, psychological, social, spiritual/religious and economic needs. In addition, antitumor treatments are complicated and long‐term in many cases and may be associated with significant challenges and side effects. Meeting these needs and managing these challenges requires teamwork and interdisciplinary care, which can only be achieved through inter‐professional communication.

A head nurse with 5 years of experience in oncology said:

Nursing colleagues with the accompaniment of doctors, psychologists and social workers should know how to communicate with children with cancer and their families. This makes families trust their child's nurses and cooperate with them from the moment the disease is diagnosed until the time of recovery or even when the child's condition is poor…. [N2]

A nurse with 6 years of work experience in the paediatric oncology department stated:

We do not have a full‐time psychologist in the oncology unit despite the need for good teamwork. The number of personnel is also insufficient. On the other hand, there is only one pediatric oncologist. [N6]

#### 3.3.2 Holding Educational Workshops

Providing high‐quality and holistic nursing care for children with cancer requires appropriate theoretical knowledge and practical skills regarding treatments, procedures, side effects, and the needs of patients and families. In addition to medical problems, nurses have to manage spiritual, religious and ethical issues when caring for children with cancer. Many of these issues and challenges are not included in nursing education courses in Iran. On the other hand, medical and nursing professions are advancing increasingly, and new medications, procedures, techniques and care methods are being introduced constantly. Thus, nurses and all healthcare professionals need continuous education to provide updated and holistic care for their clients.

A nurse with 4 years of experience in oncology expressed that:

Nursing is a combination of knowledge, skills, high‐level decision‐making ability, and management skills. They held a workshop on chemotherapy drugs and side effects for us, but no training course has been held on how to manage the religious/spiritual crisis of children and parents. We don't know how to manage these conditions… [N4]

#### 3.3.3 Training Paediatric Oncology Nurse Specialists

Paediatric oncology nursing is a sensitive and highly specialised profession with various responsibilities, such as preparing patients for chemotherapy and diagnostic and medical procedures, providing palliative care if necessary, providing psychosocial and spiritual support, and collaborating with other members of the healthcare team. This profession also requires special skills such as paediatric basic and advanced life support, prescribing and handling chemotherapeutic drugs, establishing communication with children, their families, and other healthcare professionals, and managing the unique needs of patients. Thus, training skilled nurses in paediatric oncology is crucial. However, there is no specific training course for paediatric oncology nursing in Iran, and participants in this study identified the need for such courses.

A nurse with 2 years of experience in oncology stated:

It is tough and challenging for me to see mothers and fathers who experience their child's illness. Besides caring for their child, I ask myself what other help I can do. Before, I did not have such concerns when working in the surgery unit. However, in the oncology unit, in addition to taking care of children, it is crucial to communicate with their mothers and fathers. In my opinion, training pediatric oncology nurses in the country would be very helpful because they receive specialized education. [N1]

This category highlights the importance of effective communication among nurses and other healthcare professionals to deliver holistic care to children with cancer and their families. Additionally, nurses should participate in regular educational workshops and receive specialised training in paediatric oncology.

## 4 Discussion

We explored the experiences and perceptions of nurses and parents of children with cancer regarding the importance of communication in caring for children with cancer. Three categories and eight subcategories emerged from data analysis. These categories are discussed below.

### 4.1 Category 1: Supportive Communication

The first subcategory of supportive communication was *informational support*. Although establishing effective communication with all patients is necessary, establishing such communication for children with cancer and their parents is doubly crucial. Parents of children with cancer who participated in this study reported that their cancer information was insufficient. This, in turn, may lead to an increased psychological burden for children and their parents. In this regard, Sisk, Schulz, Blazin, et al. (2021), in their qualitative study, identified six distinct functions for communication in paediatric oncology (Sisk, Schulz, Blazin, et al. 2021). One of these functions was exchanging information. Participants in that study reported needing information about their care and educational materials that address their needs. Sisk, Mack, et al. (2018) demonstrated that ‘exchanging information’ was one of the communication functions in paediatric oncology. Their results also showed that the provision of information for children with cancer and their parents is associated with positive outcomes such as feeling acknowledged, hopefulness, peace of mind, and increased trust in the healthcare team (Sisk, Mack, et al. 2018). Generally, the results mentioned above show that children with cancer and their families have significant information needs, and one of the best ways to address this need is through establishing effective communication.

The other two subcategories of supportive communication included ‘psychological support’ and ‘special communication skills’. Results of our study showed that diagnosing a child with cancer faces families with many challenges and causes the parents to spend a lot of energy due to the long and exhausting treatment and care. In many cases, the pressures caused by caring for a sick child increase to such an extent that it affects the physical, psychological and social health of parents negatively. These families need *psychological support*, and healthcare professionals (including nurses) should have *special communication skills*, such as caring with compassion and empathy, to address these families' needs. In this regard, Sisk, Mack, et al. (2018) showed that nurses and families of children with cancer consider emotional and psychological support an essential aspect of nursing care. This study also reported that the psychological and emotional needs of families of children with cancer can be met through sensitive communication and using special skills by the healthcare team, such as politeness, empathy, communication with compassion, honesty and showing emotions, which is consistent with our results. In a qualitative study in 2021, Sisk et al. reported six functions for communication in paediatric oncology, one of which was ‘addressing emotions’ with three subcategories: exploring and adapting to emotional needs, being encouraging and supportive, and respecting patient boundaries. Parents of children with cancer who participated in that study reported that they need medical care to be adapted to their emotional state, provision of encouragement and support, their boundaries to be respected, and their child's worries, concerns, and apprehension to be acknowledged (Sisk, Schulz, Blazin, et al. 2021). A systematic review study by Hentea et al. (2018) evaluated parent‐centred communication in paediatric oncology. Generally, the findings of that systematic review demonstrated that parents of children with cancer appreciate medical care providers (including nurses) who are sensitive to their needs and have special communication skills, such as being empathetic, calm, attentive, compassionate and honest. These results further emphasise providing proper psychological support for children with cancer and their families through establishing effective and sensitive communication.

The findings of our study in this category indicate that families of children with cancer in Iran have various informational and psychological needs that healthcare professionals, particularly nurses, have not fully addressed. To better support these families, nurses and other healthcare professionals should focus on establishing effective communication and employing specialised communication skills.

### 4.2 Category 2: Managing Spiritual Crisis

Results of our study showed that communicating with children with cancer and their parents should be significant and crucial for the healthcare team to give these patients a purposeful and meaningful life. The first function of communication with these families is to support them. Still, considering the special conditions of the families who have a child with cancer, the communication process should go to higher levels to have a more profound impact on families. If the communication does not go beyond the supporting stage, it will not be effective for extended periods. Therefore, one of the responsibilities of nurses who care for children with cancer and their families is spiritual management. Since Iran is a highly religious society, all healthcare professionals, including nurses, should pay special attention to the spiritual needs of children with cancer and their families. In this regard, our findings showed that healthcare providers should identify the spiritual/religious beliefs and needs of children with cancer and their parents and also manage the spiritual crisis of these families.

Some studies have evaluated the importance of spirituality and spiritual care in paediatric oncology settings. For example, Meireles et al. (2015) revealed that when healthcare providers respect the spirituality of children with cancer and their families, the communication between them becomes stronger, and their confidence increases. Respecting spirituality was also reported as a psychological support that can improve quality of life and promote engagement in treatments. Results of this study also showed that spirituality helps families of children with cancer in coping with difficult situations and helps the healthcare team support families during the paediatric cancer journey (Meireles et al. 2015). In their scoping review, Petersen (2020) demonstrated that spiritual care can provide the necessary support for parents of children with cancer and enhance their coping capabilities. They also reported that spirituality and spiritual care increase parental hope, assist them in finding meaning and purpose in life, and guide parents to improve their communication with their children. Authors of this study have suggested that paediatric oncology nurses may offer proper spiritual care through skillful communication with children with cancer and their families (Petersen 2020). Juškauskienė et al. (2023) reported that children who are diagnosed with cancer have their own opinion regarding the need to pray or not. These children also have a unique understanding of spirituality that is related to their age, gender, and family composition and changes as children develop and mature. With this in mind, Juškauskienė et al. suggest that exploring the spiritual lives of children diagnosed with life‐threatening illnesses is crucial (Juškauskienė et al. 2023). In a qualitative study in Iran, Borjalilu (2022) revealed that children with cancer experience various spiritual issues and challenges, such as philosophical questions (about life and death), faith and hope, relation with nature, love, and social support, expressing their fear, anxiety and aggression, etc. Participants in this study stated that their children frequently ask questions about why they had become sick, life and death, life after death, heaven and hell. They also reported that their children pray more often for their health (Borjalilu 2022). In another qualitative study in Iran, Reisi‐Dehkordi et al. (2014) showed that one of the main problems experienced by children with cancer and their mothers was ‘spiritual problems’ with three subthemes, including: ‘why me’, ‘connection with God’ and ‘divine punishment’. The findings of this study also revealed that children with cancer have philosophical, spiritual questions such as: why have they become sick? Why are other children not sick? Why doesn't God love them? Why doesn't God answer their prayers? Is God punishing me? Such questions were also asked by mothers of children with cancer (Reisi‐Dehkordi et al. 2014). In addition, Ahmadi Pishkuhi et al. (2018) (Ahmadi Pishkuhi et al. 2018) evaluated the experiences of parents of children with cancer. One theme that emerged in this study was ‘the inability of parents to answer their children's questions’. These parents stated that their children ask philosophical questions that are difficult to answer, such as: ‘Do I go to paradise after death?’, ‘If I die, can I still see you?’, ‘If I promise to be a good boy, I don't die anymore?’ and ‘Has God put on sickness and pain to punish my mistakes?’.

Generally, our results in this category indicate that addressing the spiritual needs of children with cancer and their families is far beyond and significantly different from merely meeting their psychological and informational needs. Thus, nurses in paediatric oncology settings, especially in Iran, which is a highly religious and spiritual society, should pay special attention to the spiritual needs and crises of families with children who have cancer and manage them by establishing proper communication.

### 4.3 Category 3: Empowering Parents and Healthcare Team

Our results showed that parents of children with cancer and healthcare professionals need to be empowered to provide the best possible care for these children. In this regard, participants' statements in our study revealed that providing high‐quality and holistic care for paediatric oncology patients and their families requires sufficient collaboration and interaction between children, families and all healthcare professionals, which can be achieved through proper communication and *teamwork*. In addition, *training paediatric oncology nurse specialists* and *holding educational workshops* for nurses and all other healthcare professionals to provide continuous and on‐the‐job education are essential strategies that can be considered by policymakers in the healthcare system. These strategies can be applied to improve the quality of medical and nursing care provided for children with cancer and their families.

Regarding the importance of multidisciplinary care and the *necessity of teamwork* in paediatric oncology settings, Gulati et al. (2014) reported that working within an interdisciplinary team has significant benefits such as ‘sharing expertise and collaboration’, ‘giving and receiving social and emotional support’ and ‘being valued by and valuing team members’. However, they have also discussed some demands of this teamwork, including: ‘interpersonal and communication tensions’, ‘conflicting views about providing care’, ‘role confusion’ and ‘overlap and being undervalued’ (Gulati et al. 2014). In their cross‐sectional survey, Graetz et al. (2023) stated that 90% of participating nurses reported daily communication with other nurses, and 66% had daily communications with oncologists. Most participants reported that interdisciplinary communication is necessary to establish treatment goals and prognosis, identify patients' preferences, and determine the first treatment modality. Results of this study also revealed that healthcare providers with more interdisciplinary collaboration and teamwork had higher job satisfaction and perceived higher quality of provided care (Graetz et al. 2023). Based on these results, it can be stated that teamwork and interdisciplinary collaboration are associated with significant benefits for children with cancer, their families, and healthcare providers. However, this collaboration and teamwork may be associated with some challenges and issues, especially in interpersonal communications, that must be appropriately managed to avoid conflicts between healthcare professionals.

Some studies have discussed the importance of *training paediatric oncology nurse specialists* and providing continuous education for them in various forms, such as *holding educational workshops*. For example, Challinor et al. (2014) demonstrated that participating nurses had education needs about chemotherapy and adverse effects, preventing infections, pain control and palliative care. They also needed education regarding diagnostic procedures in paediatric oncology, sexuality care, care of intravenous access devices, caring for adolescent patients and adverse effects of radiotherapy (Challinor et al. 2014). Enskär (2012) indicated that when paediatric oncology nurses can participate in continuous education courses and fulfil the needs of children and their families, this may enhance their possibility of becoming experts and maintaining expert competence (Enskär 2012). These results and our results demonstrate that paediatric oncology nursing is a highly specialised and advanced profession requiring special theoretical knowledge and advanced practical skills. Thus, training paediatric oncology nurse specialists and providing continuous education for them is crucial for addressing the various and complex needs of children with cancer and their families in all aspects of their lives. However, in many countries, such as Iran, there is no specific education course for training paediatric oncology nurse specialists. Furthermore, we found no study evaluating the educational needs or clinical competencies of nurses who care for children with cancer and their families in Iran. Thus, we strongly recommend that healthcare professionals, researchers and policymakers pay more attention to this subject and make their best efforts to enhance the profession of paediatric oncology nursing in Iran.

### 4.4 Study Limitations

One limitation of the present study is the generalizability of the results. We only evaluated the perceptions of nurses and parents of children with cancer, and other healthcare professionals such as oncologists, social workers, paediatricians, etc., as well as siblings of patients were not included in the study. In addition, this study was conducted in only one hospital unit, which is not specific to paediatric oncology patients; adult oncology patients are also admitted to this unit. These factors may limit the generalizability of our results for other cultural backgrounds. Moreover, participants and researchers of this study are Persian‐speaking, and some local expressions that participants have stated may not have direct English translation and may influence the interpretation of our results.

## 5 Conclusion

Informational communication is the first principle of communication in the paediatric oncology unit. Psychological support of parents is another principle of supportive communication. Because of the complex conditions of children with cancer and its effects on their parents, nurses in the oncology unit should pay special attention to psychological support in communication with these parents. Of course, parents need informational and communication support and experience spiritual crises; thus, nurses need special communication skills to provide effective communication to parents of children with cancer. It is also essential to note that optimal communication in the paediatric oncology unit can be achieved through proper teamwork and sufficient staff. Health managers should pay special attention to holding periodic workshops for training nursing and medical professionals. Furthermore, health authorities should develop special educational courses for paediatric oncology nurses.

## 6 Relevance to Clinical Practice and Future Research

All healthcare providers (including nurses, oncologists, paediatricians, etc.) can consider the results of this study to improve their communications with other healthcare team members, children with cancer and their families to increase the quality of care they provide.

Healthcare researchers, educators and policymakers, especially in Iran, should pay more attention to the field of paediatric oncology nursing and develop and evaluate various strategies and interventions for improving communication between healthcare professionals, children with cancer and their families. Also, evaluating the care needs of these patients in different aspects of life, as well as the educational needs of the healthcare team, and taking proper measures for addressing these needs should be noted.

## Author Contributions

Study conception and design: Haydeh Heidari and Reza Saidi. Data collection and data analysis: Haydeh Heidari. Drafting the manuscript: Reza Saidi. Review and editing the manuscript: Haydeh Heidari. Both authors have read the final manuscript and approved its content.

## Ethics Statement

This study is approved by the ethics committee of Shahrekord University of Medical Sciences (ethics code: IR.SKUMS.REC.1402.069).

## Conflicts of Interest

The authors declare no conflicts of interest.

## Data Availability

The data that support the findings of this study are available from the corresponding author upon reasonable request.
